# Simultaneous achievement of high ethanol yield and titer in *Clostridium thermocellum*

**DOI:** 10.1186/s13068-016-0528-8

**Published:** 2016-06-02

**Authors:** Liang Tian, Beth Papanek, Daniel G. Olson, Thomas Rydzak, Evert K. Holwerda, Tianyong Zheng, Jilai Zhou, Marybeth Maloney, Nannan Jiang, Richard J. Giannone, Robert L. Hettich, Adam M. Guss, Lee R. Lynd

**Affiliations:** Thayer School of Engineering, Dartmouth College, 14 Engineering Drive, Hanover, NH 03755 USA; Bredesen Center, University of Tennessee, Knoxville, TN 37996 USA; Biosciences Division, Oak Ridge National Laboratory, Oak Ridge, TN 37831 USA; Bioenergy Science Center, Oak Ridge National Laboratory, Oak Ridge, TN 37831 USA; Chemical Sciences Division, Oak Ridge National Laboratory, Oak Ridge, TN 37831 USA

**Keywords:** Consolidated bioprocessing, *Clostridium thermocellum*, Cellulosic ethanol, Adaptive evolution

## Abstract

**Background:**

Biofuel production from plant cell walls offers the potential for sustainable and economically attractive alternatives to petroleum-based products. Fuels from cellulosic biomass are particularly promising, but would benefit from lower processing costs. *Clostridium thermocellum* can rapidly solubilize and ferment cellulosic biomass, making it a promising candidate microorganism for consolidated bioprocessing for biofuel production, but increases in product yield and titer are still needed.

**Results:**

Here, we started with an engineered *C. thermocellum* strain where the central metabolic pathways to products other than ethanol had been deleted. After two stages of adaptive evolution, an evolved strain was selected with improved yield and titer. On chemically defined medium with crystalline cellulose as substrate, the evolved strain produced 22.4 ± 1.4 g/L ethanol from 60 g/L cellulose. The resulting yield was about 0.39 g_ETOH_/g_Gluc eq_, which is 75 % of the maximum theoretical yield. Genome resequencing, proteomics, and biochemical analysis were used to examine differences between the original and evolved strains.

**Conclusions:**

A two step selection method successfully improved the ethanol yield and the titer. This evolved strain has the highest ethanol yield and titer reported to date for *C. thermocellum*, and is an important step in the development of this microbe for industrial applications.

**Electronic supplementary material:**

The online version of this article (doi:10.1186/s13068-016-0528-8) contains supplementary material, which is available to authorized users.

## Background

Low-carbon liquid fuels and organic chemicals will likely be derived from plant biomass, of which lignocellulose is the most prominent component. An economic process to overcome the recalcitrance of cellulosic biomass will lead to more widespread utilization of this resource [1]. Consolidated bioprocessing (CBP), in which biomass solubilization and fermentation are accomplished in one step without added enzymes, is a promising configuration for low-cost biological conversion of plant cell walls [2, 3]. Key factors that determine the economic viability of CBP, as with any chemical transformation, are yield, titer, and rate. Target performance metrics for cost-effective production of ethanol from lignocellulose are a yield of >90 % of theoretical, titer of >40 g/L, and rate of 1 g/L/h [4].

**Fig. 1:**
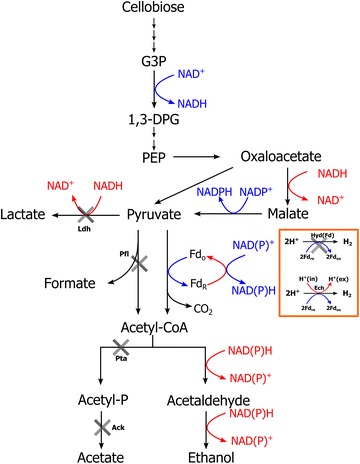
The ethanol pathway in *C. thermocellum* strain AG553. *Gray crosses* represent gene deletions. NAD(P)H producing pathways are in *blue*, and NAD(P)H consuming steps are in *red*

*Clostridium thermocellum* is a good candidate organism for CBP due to its ability to rapidly ferment cellulose and produce ethanol. Wild-type strains typically produce ethanol with a yield of about 10–35 % of the theoretical maximum [5]. Under controlled fermentation conditions, 100 g/L cellulose can be converted to 15 g/L ethanol in 75 h [6]. Several approaches have been pursued aimed at engineering *C. thermocellum* to produce ethanol at higher yield [5], including mutations that block acetate, lactate [7], H_2_ [8], and formate [9] production. The most successful of these to date involved simultaneous deletion of *hpt*, *hydG*, *ldh*, *pfl*, and *pta*-*ack* [10]. The resulting pathway for conversion of cellobiose to ethanol is redox balanced (Fig. 1). This strain, AG553, produced ethanol at 63.5 % of the theoretical maximum yield from 5 g/L cellulose, with lower yields observed at higher initial cellulose concentrations. Decreasing yield with increasing titer has also been observed for the wild-type strain [6], as well as other engineered strains of *C. thermocellum* [7, 11]. The highest reported titer produced by *C. thermocellum* is 23.6 g/L, although this was for strain I-1-B, an environmental isolate from a hot spring in Japan which is not widely available [12]. Growth of wild-type *C. thermocellum* is inhibited by ethanol at concentrations of 4–16 g/L [1]; however, there are several reports showing that this organism can be adapted to grow in the presence of 50 g/L ethanol by serial transfer [13, 14].

To increase yield and titer, we started with strain AG553, using an adaptive evolution strategy to enrich for strains with faster growth in the hope that, due to the constraints on metabolism from gene deletions, ethanol production would improve. After adaptation, the resulting strains were characterized by whole genome sequencing as well as physiological, proteomic, and biochemical approaches to better understand the processes behind improved ethanol production.

## Results and discussion

### First round of adaptive evolution

**Fig. 2:**
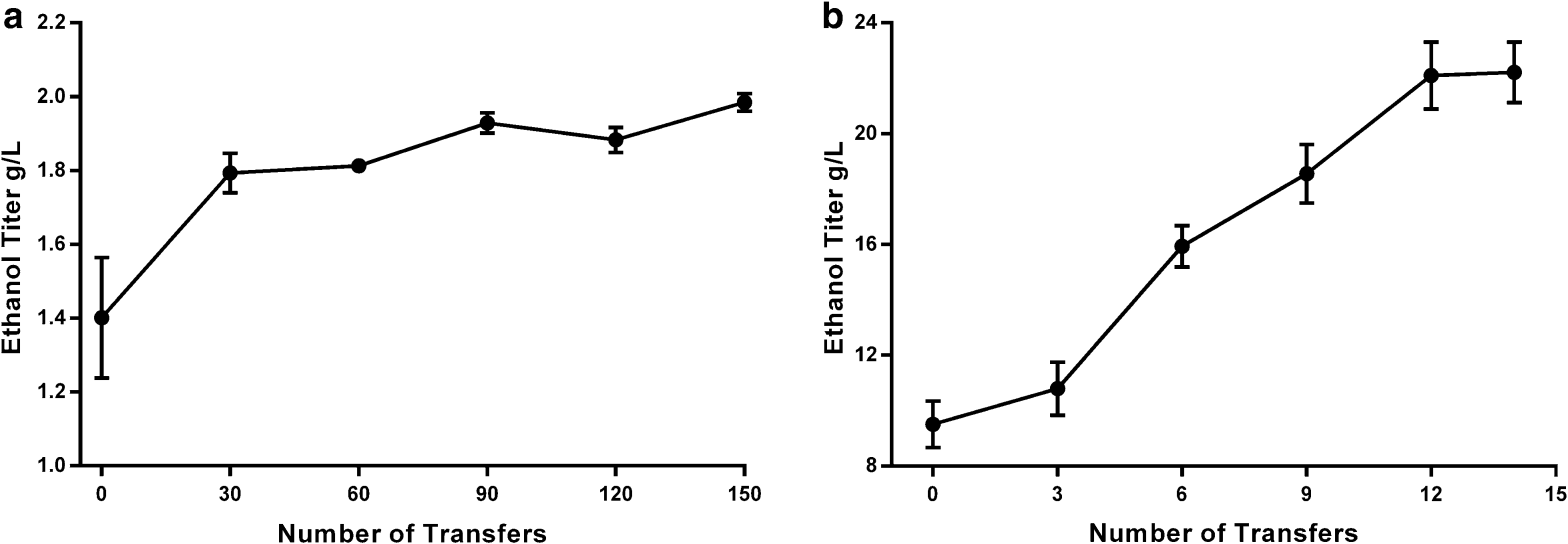
Ethanol production during the course of strain evolution. **a** First round of evolution, ethanol titer determined from 5 g/L cellobiose as substrate in CTFUD medium. **b** Second round of selection, ethanol titer determined from 50 g/L cellobiose as substrate in MTC medium

*Clostridium thermocellum* strain AG553 grows much more slowly than wild type [10], presumably due to metabolic bottlenecks or imbalances. To improve the growth rate, the strain was serially transferred approximately daily in rich medium (CTFUD) with 5 g/L cellobiose, such that faster growing cells would begin to dominate the culture. Each transfer consisted of a ~1000-fold dilution, or approximately 10 generations. After 150 transfers (~1500 generations), the increase in ethanol production had slowed (Fig. 2a), and growth rate had significantly improved (Table 1).

**Table 1:** Comparison of strains grown on low substrate (5 g/L cellobiose)

Strain name	Description	Growth rate μ (h^−1^)
AG553	*C. thermocellum* DSM1313 *Δhpt ΔhydG Δldh Δpfl Δpta*-*ack* [10]	0.06 ± 0.01
AG601	Selected from AG553 after first stage adaptive evolution	0.10 ± 0.01
LL1210	Selected from AG601 after second stage adaptive evolution	0.22 ± 0.02

Error bars represent one standard deviation, *n* = 3

The nature of this type of laboratory evolution results in the cultures being a mixture of genetic backgrounds, as different cells within the population acquire different mutations. This diversity of genotypes makes the understanding of the causative mutation difficult and further genetic manipulation impossible. Therefore, the isolation of an individual genetic background strain was necessary. An individual member of the population was isolated via single colony purification after transfer #150 and named AG601.

To determine the effect on ethanol titer, AG601 was grown in minimal medium with increasing substrate concentration from 5 to 50 g/L. However, preliminary experiments showed a decrease in yield as substrate loading increased (Additional file 1). Therefore, we performed a second round of adaptive evolution with a higher initial substrate concentration.

### Second round of adaptive evolution

In this second round of adaptive evolution, we increased the substrate concentration to 50 g/L and switched from a rich medium (CTFUD) to a defined medium (MTC5) to avoid potential problems with auxotrophy and more closely mimic industrial conditions. The strain was transferred once per week, and ethanol titer was measured every 3rd transfer (Fig. 2b). After 13 serial transfers (~1000 generations total), the ethanol titer had increased from 9.5 ± 0.8 to 22.1 ± 1.2 g/L. This culture was used for single colony purification to isolate a pure genetic background, and one isolate was named LL1210.

### Comparison of evolved strains on cellobiose

To examine strain improvements via evolution, the wild type and resulting three strains (AG553, AG601, and LL1210) were cultivated in serum bottles in defined medium. Maximum growth rate was determined on 5 g/L cellobiose (Table 1). The wild-type strain had the fastest growth rate, while the growth rate of the unevolved strain AG553 was the slowest. Each round of selection resulted in a strain with a faster growth rate than the previous culture.

**Fig. 3:**
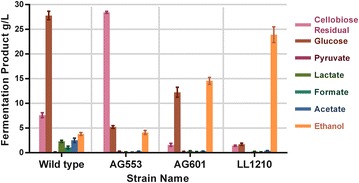
Serum bottle batch fermentation products of *C. thermocellum* from 50 g/L cellobiose. Strains were grown on minimal medium with 50 g/L cellobiose in serum bottle. *Error bars* represent one standard deviation, *n* = 3

Fermentation products were then measured after growth in batch culture in serum bottles on 50 g/L cellobiose (Fig. 3). Wild-type *C. thermocellum* had low ethanol yield and titer and produced large quantities of glucose. This is evidence of metabolic inhibition, where enzymatic hydrolysis of cellobiose continues even after *C. thermocellum* sugar catabolism stops. The unevolved strain AG553 consumed very little cellobiose, so even though the yield was higher than the wild-type strain, the titer was similar to that of wild type. The first round of selection (from AG553 to AG601) resulted in dramatically improved cellobiose consumption, and improved ethanol production, but about half of the cellobiose was still being converted to glucose. The second round of selection (from AG601 to LL1210) showed further improvements in ethanol production as well as reduced accumulation of glucose. Compared to the unevolved strain (AG553), strain LL1210 showed a 5.8-fold higher ethanol titer.

### Batch culture of final evolved strain (LL1210) grown on cellulose

**Fig. 4:**
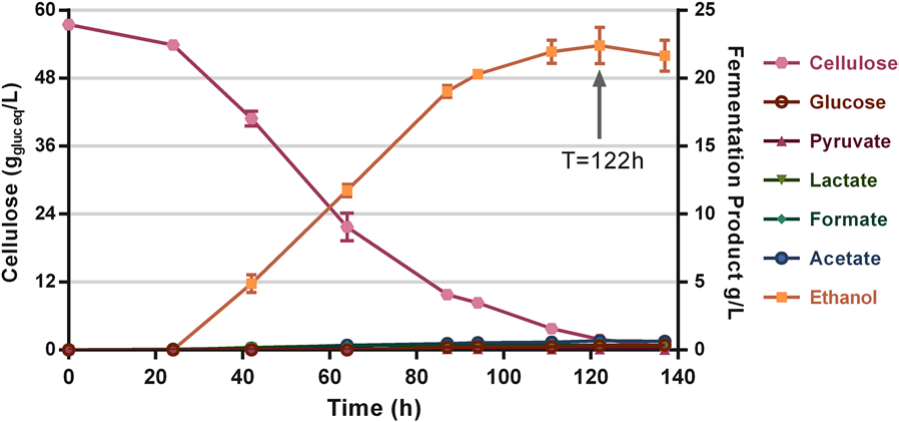
Residual substrate and product concentrations from 60 g/L from cellulose fermentation in a bioreactor. The strains grown on minimal medium in a bioreactor with pH regulation. *Error bars* represent one standard deviation, *n* = 3

*Clostridium thermocellum* is noted for its ability to rapidly consume cellulose [15], and we wanted to confirm that we had not impacted this ability during selection. In a bioreactor batch fermentation of 60 g/L cellulose (Avicel PH105), 95 % of the substrate was consumed (Fig. 4), with a final ethanol titer of 22.4 ± 1.4 g/L and yield of 0.39 g_EtOH_/g_Glu eq_, which is 75 % of the maximum theoretical yield. The production of by-products including pyruvate, lactate, formate, and acetate were all less than 0.5 g/L. The cellulose consumption rate was 1.4 ± 0.2 g/L/h which is close to the cellobiose consumption rate of 1.6 ± 0.3 g/L/h.

The carbon recovery based on the *T* = 122 h sample was calculated, and the total carbon recovery was 99.8 % (Table 2). Besides biomass (7.2 %), the main by-product of the fermentation was extracellular amino acids which accounted for 10.1 % of the carbon. Valine (1.63 ± 0.18 g/L), alanine (0.81 ± 0.08 g/L), glutamate (0.70 ± 0.07 g/L), and threonine (0.69 ± 0.10 g/L) were the most abundant amino acids. Together, this demonstrates that LL1210 has retained the ability to efficiently utilize cellulose as a carbon and energy source.

**Table 2:** Carbon balance from fermentation of 60 g/L cellulose (345 mM glucose equivalents)

Compound	mM	% C3 (pyruvate)^a^
Ethanol	520.9	75.5
Ex amino acid carbon^b^	208.2	10.1
Biomass carbon	148.3	7.2
Ex protein carbon^c^	50.8	2.1
Acetate	11.9	1.7
Ex sugar^d^	10.4	1.5
Isobutanol	3.5	1.0
Malate	3.0	0.4
Lactate	2.0	0.3
Glucose	0.6	0.1
Total		99.8

^a^To facilitate comparison, carbon-containing compounds were expressed in terms of C3 equivalents. For example, one C3 equivalent (i.e., pyruvate) is required to produce one ethanol

^b^Ex amino acid carbon; amount of carbon in extracellular free amino acids

^c^Ex protein carbon; amount of carbon in extracellular (secreted) protein

^d^Ex sugar; extracellular sugar, including all the soluble glucan and xylan

### Ethanol tolerance

**Fig. 5:**
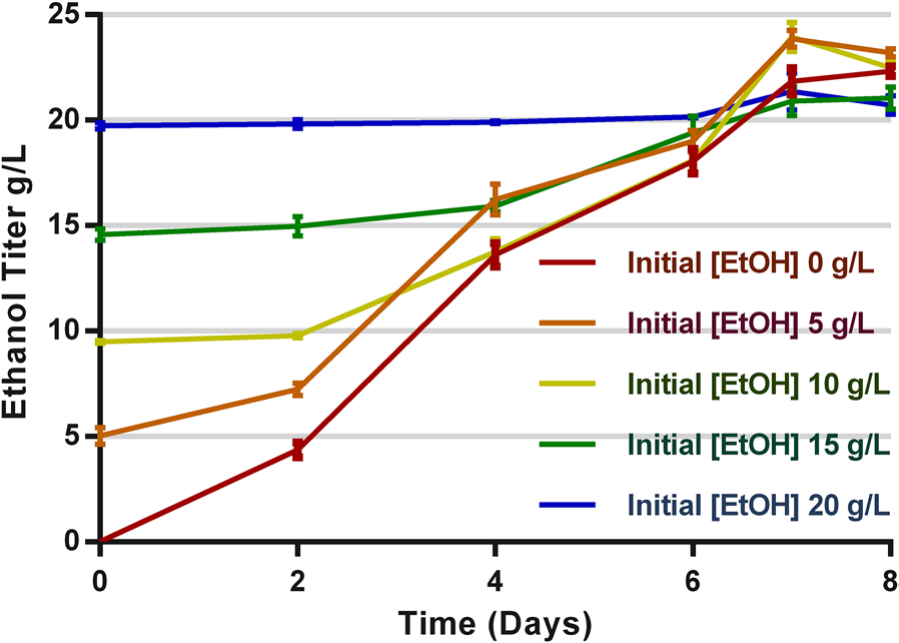
Batch fermentation *C. thermocellum* LL1210 with different initial ethanol concentrations. The strains grown on minimal medium with 50 g/L cellobiose in serum bottles. *Error bars* represent one standard deviation, *n* = 3

To try to reach even higher titers, strain LL1210 was further grown on 95 g/L Avicel in a bioreactor, achieving 98 % solubilization. The ethanol titer increased from 22.4 ± 1.4 to 26.7 ± 0.9 g/L, but the yield decreased to 0.29 g_ETOH_/g_Gluc eq_ (Additional file 2). The fact that the titer from 95 g/L cellulose was similar to the titer from 60 g/L cellulose suggested that the decrease in yield might be due to inhibition by ethanol. To test this hypothesis, strain LL1210 was grown in serum bottle batch cultures with different initial ethanol concentrations from 0 to 20 g/L (Fig. 5). Regardless of the initial ethanol concentration, the final concentration was around 22 g/L, suggesting that ethanol tolerance is currently limiting the ethanol titer in this strain.

### Identification of the genes responsible for LL1210 strain adaptive evolution

The key phenotypes we observed in the evolved strains were improved growth rate, reduced glucose accumulation, improved cellobiose consumption, and improved ethanol production. To better understand the genetic basis for these changes, we sequenced the genomes of strains AG553, AG601, and LL1210 and compared them with the wild-type strain (Additional file 3).

**Fig. 6:**
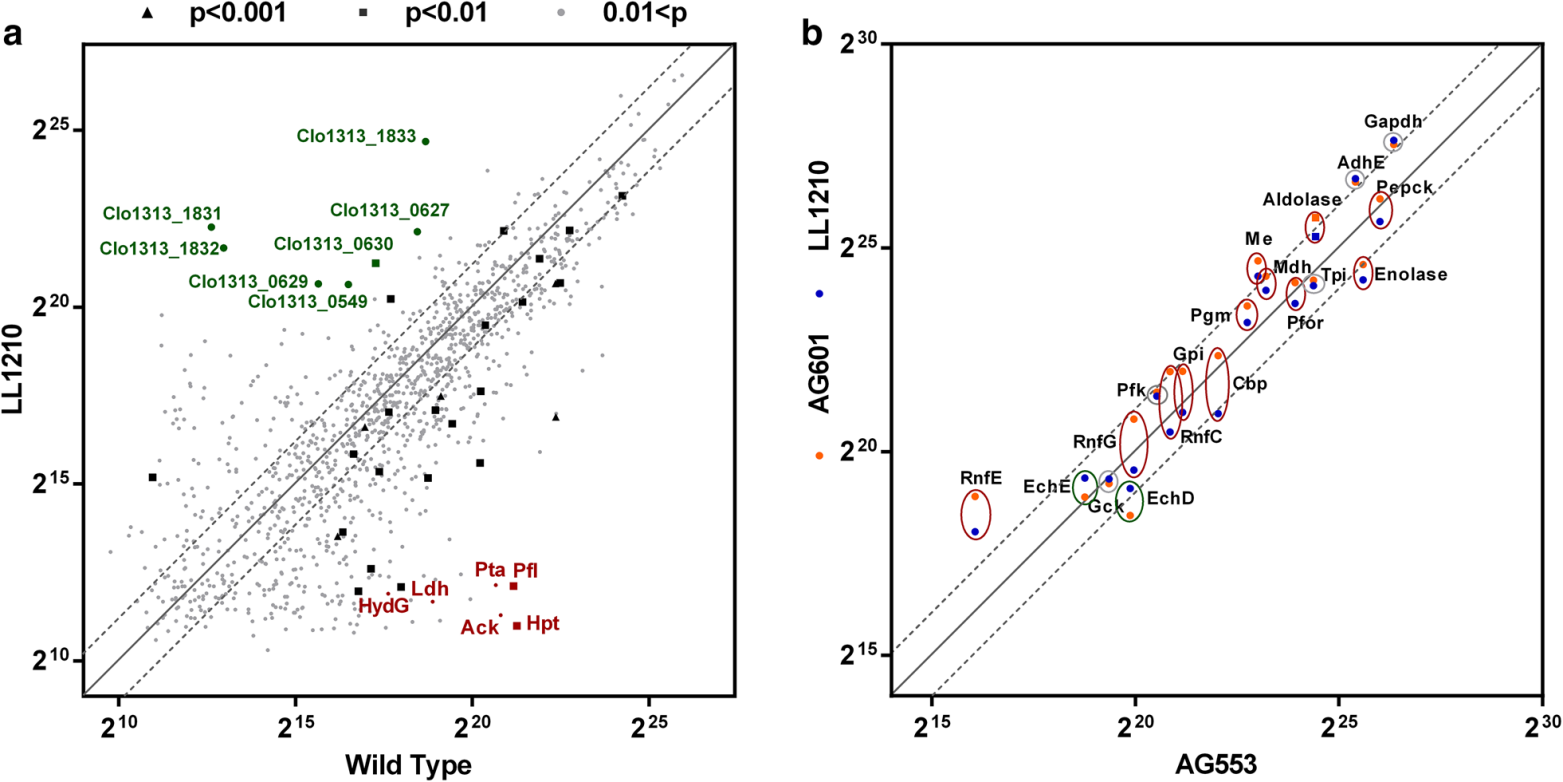
Relative protein abundance. Abundance was determined based on matched-ion intensity (MIT) and is reported in arbitrary units. Proteins that were not detected were plotted with a low-level log-normal distribution value. Each *point* represents the average of two biological replicates. For panel **a**, all the gene deletions proteins are in *red color*, and some significantly upregulated genes are noted in *green color*. For panel **b**, proteins were included for selection that are thought to play a role in central metabolism, electron transfer, and ethanol production. *Red ovals* indicate the abundances decreased significantly between AG601 and LL1210; *Green ovals* indicate the abundances increase significantly; *Gray ovals* indicated no significant difference in the abundances. The* solid diagonal line* represents a 1:1 correspondence in protein abundance between target and control strains. The *dashed lines* indicate twofold changes in abundance

All of the targeted genes for deletions (*ldh, pta*-*ack, pfl, hydG*, and *hpt*) were absent in AG553 and its descendants, as expected. During normal strain construction, mutations that spontaneously occur within the strain become fixed during single colony purification. Strain AG553, which was not intentionally evolved, contained a single nucleotide variation (SNV) in the bifunctional alcohol (ADH) and aldehyde dehydrogenase (ALDH), *adhE* (Clo1313_1798). This SNV originated in strain LL350 (*∆hpt∆hydG*) [8], which is part of the AG553 lineage, and it increases NADPH-dependent ADH activity [16]. A mutation also occurred 114 bp upstream of the *ech* hydrogenase gene (Clo1313_0575) (Additional file 3). We subsequently saw an increase in abundance of some of the Ech protein subunits (Clo1313_0571-2) (Additional file 4) in proteomics studies described below, suggesting that this SNV may increase *ech* expression. Clo1313_1831 is annotated as a “ROK domain containing protein” and contains a helix-turn-helix domain and a sugar kinase domain. The presence of a ROK domain often indicates function as a transcriptional repressor [17]. These mutations may be responsible for the observed change in the abundance of proteins Clo1313_1831-3 (Additional file 4; Fig. 6a).

During the evolution from strain AG553 to AG601, a number of mutations occurred in or near genes related to the stress response and could be related to the improvement in strain performance. One such mutation was in the region upstream of DnaK (Clo1313_0933), a chaperone involved in protein folding and the stress response [18]. One might expect this mutation to alter expression of *dnaK*; however, there was no significant change in DnaK levels (see proteomics data below), suggesting that this mutation might be neutral. Another mutation introduces an amino acid change in one of the two homologs of Spo0A (Clo1313_0637), typically the master regulator of sporulation. While most spore-forming bacteria have a single homolog of *spo0A*, *C. thermocellum* has two, Clo1313_0637 and Clo1313_1409, which share 56 % amino acid identity with each other. The Clo1313_1409 protein is 58.3 % identical to the well-characterized *Bacillus subtilis* Spo0A, whereas Clo1313_0637 is only 49 % identical. Previous deletion of Clo1313_1409 completely eliminated sporulation in *C. thermocellum* [19], suggesting that it might be the primary controller of sporulation. Interestingly, a mutant strain of *C. thermocellum* ATCC27405 that was evolved to be tolerant to poplar hydrolysate was found to have a mutation in the second *spo0A* homolog Cthe_3087 [20] (99 % amino acid identity to Clo1313_0637), and we also found a mutation in this gene, suggesting that the second Spo0A may also play a role in coordinating a stress response, such as sporulation. This mutation could allow the cell to continue to metabolize sugar and grow even under stressful conditions. Further investigation into the role of Clo1313_0637 may help elucidate its function. Another potentially important mutation in AG601 is in the termination factor *rho* (Clo1313_2832). While this gene is typically essential for growth, SNVs in *rho* in *Escherichia coli* helped to confer resistance to ethanol by enhancing transcriptional read-through and altering gene expression [21]. A similar mechanism may be at play here. Interestingly, two mutations were identified in CRISPR/Cas genes Cas2 and Cas4, which are both predicted to be involved in the acquisition of new CRISPR spacers to confer resistance to new foreign DNA, though the relation of these mutations to improved fermentation performance, if any, is unknown.

Additional mutations occurred during the evolution from strain AG601 to LL1210. One such mutation is in *spoIIR* (Clo1313_2594). During sporulation in *Bacillus subtilis*, SpoIIR is a regulatory protein that is required for activation of SigE in the mother cell, eventually leading to mother cell lysis [22]. In strain LL1210, this mutation could help prevent metabolic shutdown, allowing continued fermentation of sugars even under stressful conditions. Additionally, homologs of the *Bacillus subtilis* mRNA-processing genes ribonuclease Y (Clo1313_1122-1123; *ymdAB*) could alter mRNA stability, leading to altered protein production and perhaps a more robust stress response or better metabolic flux.

### Proteomic and enzymatic profile comparisons between original and evolved strains

To understand how adaptation affected protein expression, proteomic analysis was performed on all samples (Additional file 4). Protein abundance was compared between the wild type and final evolved strain LL1210 (Fig. 6a). Near the bottom horizontal axis, a serial of points represents proteins present in wild type, but absent or expressed at low level in strain LL1210. The most abundant of these are, in fact, the targeted gene deletions. Near the left vertical axis, another serial of points represents proteins present in LL1210, but absent or expressed at low level in wild type. Two of the most interesting proteins in this group are Clo1313_1831 and Clo1313_1832. Clo1313_1831 is the ROK-domain protein described above, and Clo1313_1832 is annotated as a phosphofructokinase (Pfk) B-domain protein, suggesting that it may be involved in conversion of fructose 6-phosphate to fructose 1, 6-bisphosphate during glycolysis. Indeed, PFK activity is increased in the evolved strains (Table 3), which might explain their improved growth.

**Table 3:** Enzyme activity comparison

Strain name	PFK activity^a^	ALDH activity		ADH activity		GAPDH activity
	PPi	NADH	NADPH	NADH	NADPH	NADH
Wild type	1.60 ± 0.40^b^	N/A	N/A	12.53 ± 2.59	N/A	0.38 ± 0.09
AG553	2.31 ± 0.54	1.32 ± 0.40	0.00 ± 0.06^c^	7.67 ± 1.19	6.82 ± 0.16	0.28 ± 0.07
AG601	2.84 ± 0.37	1.10 ± 0.17	0.00 ± 0.14	8.63 ± 0.74	6.74 ± 0.57	0.40 ± 0.11
LL1210	4.45 ± 0.81	2.92 ± 0.89	0.02 ± 0.11	14.88 ± 2.33	14.9 ± 0.31	0.53 ± 0.15

^a^Activity units are in U/mg protein

^b^Measured in triplicates with same sample at different concentrations. Error represents one standard deviation, *n* = 3

^c^When background activity was higher than reaction activity, the values are reported as zero

Interestingly, several cellulosomal proteins also show increased expression (Fig. 6a), including CipA (Clo1313_0627), OlpB (Clo1313_0628), Orf2 (Clo1313_0629), and OlpA (also called SdbA, Clo1313_0630). These proteins encode the primary and secondary scaffoldin components of the cellulosome and have been shown to play a key role in cellulose consumption [23–25]. It is possible that the increased abundance of these proteins plays a role in the ability of the evolved strains to consume high concentrations of cellulose.

To understand how changes in protein abundance may have affected metabolism, we selected a subset of proteins thought to participate in glycolysis, electron transfer, and ethanol production, and compared their abundance in the two evolved strains (AG601 and LL1210) against their abundance in the parent strain (AG553) (Fig. 6b). In general, these metabolic enzymes exhibited less variation compared with the complete set of proteins (Fig. 6a, b). Two proteins, in particular, AdhE (Clo1313_1798) and GapDH (Clo1313_2095), are notable. Both exhibit a twofold increase despite the fact that they are among the most highly abundant proteins to begin with. AdhE is a bifunctional alcohol and aldehyde dehydrogenase [16]. In accordance with the proteomic measurements, alcohol dehydrogenase (ADH) activity was assessed and also found to have increased about twofold compared with the parent strain (Table 3). High levels of ALDH and ADH activity may be directly linked to the improvements in ethanol titer in these strains. High level of GAPDH activity may have a similar effect to the increased levels of PFK, allowing greater flux through glycolysis and therefore better ethanol production.

Another interesting observation is that many proteins whose abundance increased after the first round of selection (strain AG601) subsequently decreased after the second round of selection (strain LL1210). These cases are indicated with red colored circles. Expression of metabolic enzymes needs to be balanced for optimal flux and redox balancing [26], so the decrease in protein abundance may indicate optimization of these central metabolic pathways.

## Conclusions

In this work, an engineered strain of *C. thermocellum* strain was selected for improved growth. This selection resulted in strains with improvements in both ethanol yield and titer. The final evolved strain produced ethanol at 75 % of the maximum theoretical yield and titer of 22.4 g/L. The main products produced other than ethanol and cells were amino acids. If the carbon and electron flux currently used for amino acid production was instead directed toward ethanol production, we would expect the yield to reach 85 % of the theoretical maximum. The most apparent changes in the adapted strain are in genes Clo1313_1831-2, AdhE, and GapDH, and mutations related to sporulation and transcription. Future experiments may help elucidate the relative importance of these changes. Based on ethanol addition experiments, ethanol titer seems to be limited currently by low ethanol tolerance. Thus, more significant gains in ethanol production might benefit from adaptive selection for improved ethanol tolerance.

## Methods

### Bacterial strains, media and cultivation

Strains used in this study are listed in Table 1. All chemicals were reagent grade and obtained from Sigma-Aldrich (St. Louis, MO) or Fisher Scientific (Pittsburgh, PA) unless indicated otherwise. CTFUD rich medium [27] and MTC5 defined medium [28] were used for routine strain maintenance, strain evolution, and fermentation as indicated.

Serum bottle batch cultures were incubated at 55 °C and shaken at 180 rpm. Serum bottles were purged with N_2_ and sealed with butyl rubber stoppers. There is no gas exit or pH regulation for serum bottle batch culture. Bioreactor fermentations were carried out in 1.5-L (1-L working volume) Sartorius Biostat A-plus Sartorius Stedim (Sartorius Stedim, Bohemia, NY) bioreactors in modified MTC5 medium without MOPS buffer and with 2 g/L urea as the nitrogen source, with the temperature maintained at 55 °C and stirred at 150 rpm. The pH was controlled at 7.0 with a Mettler-Toledo pH probe (Columbus, OH) by the addition of 8N KOH. For vitamin supplementation, aliquots of a 50-fold concentrated vitamin-solution were added freshly. The bioreactor was inoculated with 5 mL −80 °C freezer stock culture grown on 5 g/L Avicel PH105 in MTC (0.5 % v/v). The headspace of the bioreactor was flushed with N_2_ gas prior to inoculation. The 95 g/L Avicel bioreactor fermentation was performed as previously described [6]. 16S rRNA gene sequences of cell pellets from each fermentation were used to verify culture purity.

### Strain evolution

For the first round of selection, strain AG553 was inoculated into 5 mL of CTFUD medium with 5 g/L cellobiose as the carbon source in a COY anaerobic chamber (COY Labs, Grass Lake, MI) with an atmosphere of (85 % N_2_, 10 % CO_2_, 5 % H_2_) at 51 °C. The culture was diluted 1000-fold (0.1 % inoculum) by transferring to fresh CTFUD medium, which was performed approximately daily for 150 transfers. Frozen stocks were stored at −80 °C after every 10 transfers. After 150 transfers, the culture was streaked on an agar plate to isolate a single colony from the evolved population. Colonies were visible and distinct after 2 days, and single colonies were selected into 5-mL CTUFD medium. Strain AG601 was one of these isolated colonies.

MTC5 chemically defined medium was used for the second round of selection. For second round of selection, all the experiments were performed in a COY anaerobic chamber (85 % N_2_, 10 % CO_2_, and 5 % H_2_) at 55 °C. Strain AG601 was firstly plated in MTC agar. 50 colonies were selected from the plate and then grown in liquid MTC medium. Their growth curves were measured by BioTek PowerWave XS plate reader (BioTek Instruments Inc., Winooski, VT). The top 20 fastest growing colonies were selected and grown in MTC medium with 50 g/L cellobiose, respectively. The cultures were transferred every week, and the inoculum was diluted ~1000-fold for a total of 13 transfers. Frozen stocks were stored at −80 °C after 12 transfers, and the culture was streaked on an agar plate to isolate a single colony from the evolved population.

### Analytical methods

Acetate, formate, ethanol, glucose, and residual cellobiose were determined by high pressure liquid chromatography (HPLC, Waters, Milford, MA) with refractive index detection using an Aminex HPX-87H column (Bio-Rad, Hercules, CA) with a 5-mM sulfuric acid solution eluent. Pellet nitrogen was determined using a Shimadzu TOC-VCPH total organic carbon analyzer with added total nitrogen unit (Shimadzu Scientific Instruments, Columbia, MD), calibrated using an acidified glycine standard [29]. 1 mL samples were centrifuged at 15,000×*g* for 10 min, the supernatant was discarded, and the pellet was rinsed twice using equal volumes of MilliQ water. Residual Avicel PH105 concentration was quantified by quantitative saccharification as previously described [6]. Based on that, Avicel concentration was converted to equivalent glucose concentration, and the ethanol yield was calculated based on the equivalents of glucose. Supernatant protein was determined with the Bradford assay (Thermo Scientific, Rockford, IL) with bovine serum albumin (BSA) (Thermo Scientific, Rockford, IL) as a standard. Secreted amino acids were measured using post-column derivatization with ninhydrin followed by separation and quantification with an Aracus Amino Acid Analyzer (membraPure, Berlin, Germany) using a T111 Li-cation exchange column as previously described [9].

### Enzyme assays

*Clostridium thermocellum* cells were harvested by centrifugation at 6000×*g* for 10 min, the supernatant was decanted, and the pellet was anaerobically suspended in a suitable buffer (dependent on assay). The cells were lysed with Ready-Lyse Lysozyme (Epicentre), and DNase I (New England Biolabs) was added to reduce viscosity. The resulting solution was centrifuged at 10,000×*g* for 5 min at room temperature, and the supernatant was used as cell-free-extract for enzyme assays.

Enzymes were assayed in an anaerobic chamber (COY Labs, Grass Lake, MI) at 55 °C with a headspace of 85 % N_2_, 10 % CO_2_, and 5 % H_2_ using an Agilent 8453 spectrophotometer. The reaction volume was 1 ml, in reduced-volume quartz cuvettes (part number 29MES10; Precision Cells Inc., NY) with a 1.0-cm path length. All enzyme activities are expressed as μmol of product min^−1^ (mg of cell extract protein)^−1^. For each enzyme assay, at least three concentrations of cell extracts were used to confirm that specific activity was proportional to the amount of extract added. Protein concentration was determined using the Bradford method with BSA as the standard.

ADH (EC 1.1.1.1 and EC 1.1.1.2) and ALDH (EC 1.2.1.3 and EC 1.2.1.3) activities were measured based on previously described methods [16], and the oxidation of NAD(P)H was observed at 340 nm (*ε* = 6.2 mM-1 cm-1). Phosphofructokinase (EC 2.7.1.11 or EC 2.7.1.90) was assayed by the oxidation of NADH, and the method was described in previous work [30]. GAPDH (EC 1.2.1.12) was measured by BioVision (BioVision Inc., Milpitas, CA) GAPDH Activity Assay Kit.

### Carbon balance calculations

Carbon balances were calculated as described previously [31]. The molar concentration of Avicel PH105 was calculated based on glucose monomers with a formula weight of 163 g/mole and 2 C3 equivalents. For glucose, extracellular sugar (non-glucose), and isobutanol, each mole was assumed to be equivalent to 2 C3 units. Ethanol, acetate, malate, and lactate were assumed to be equivalent to 1 C3 unit. For amino acids, the number of C3 units was based on calculations from Stephanopoulos et al. [32]. Alanine (1 C3 equivalent) and valine (2 C3 equivalents) account for the majority of the amino acid carbon. For biomass, 1 mol of pellet carbon was assumed to be equivalent to 1/3 mol of C3 units and based upon measured pellet nitrogen values [29]. Extracellular protein was converted to C3 equivalents by assuming that extracellular protein is 45 % carbon by mass.

### Genome-scale sequencing

Genome resequencing was performed as previously described [33]. Briefly, genomic DNA was submitted to the Joint Genome Institute (JGI) for sequencing with an Illumina MiSeq instrument.

Unamplified libraries were generated using a modified version of Illumina’s standard protocol. 100 ng of DNA was sheared to 500 bp using a focused ultrasonicator (Covaris). The sheared DNA fragments were size selected using SPRI beads (Beckman Coulter). The selected fragments were then end repaired, A tailed, and ligated to Illumina compatible adapters (IDT, Inc) using KAPA-Illumina library creation kit (KAPA Biosystems). Libraries were quantified using KAPA Biosystem’s next-generation sequencing library qPCR kit and run on a Roche LightCycler 480 real-time PCR instrument. The quantified libraries were then multiplexed into pools for sequencing. The pools were loaded and sequenced on the Illumina MiSeq sequencing platform utilizing a MiSeq Reagent Kit v2 (300 cycle) following a 2 × 150 indexed run recipe.

Paired-end reads were generated, with an average read length of 150 bp and paired distance of 500 bp. Raw data were analyzed using CLC Genomics Workbench, version 8.5 (Qiagen, USA). Reads were mapped to the reference genome (NC_017992). Mapping was improved by two rounds of local realignment. The CLC probabilistic variant detection algorithm was used to determine small mutations (single and multiple nucleotide polymorphisms, short insertions, and short deletions). Variants occurring in less than 90 % of the reads and variants that were identical to those of the wild-type strain (i.e., due to errors in the reference sequence) were filtered out. The fraction of the reads containing the mutation is shown in Additional file 3. To determine larger mutations, the CLC indel and structural variant algorithm was run. This tool analyzes unaligned ends of reads and annotates regions where a structural variation may have occurred, which are called breakpoints. Since the read length averaged 150 bp and the minimum mapping fraction was 0.5, a breakpoint can have up to 75 bp of sequence data. The resulting breakpoints were filtered to eliminate those with fewer than ten reads or less than 20 % “not perfectly matched.” The breakpoint sequence was searched with the basic local alignment search tool (BLAST) algorithm [34] for similarity to known sequences. Pairs of matching left and right breakpoints were considered evidence for structural variations, such as transposon insertions and gene deletions. The fraction of the reads supporting the mutation (left and right breakpoints averaged) is presented in Additional file 3. Raw data are available from the JGI Sequence Read Archive. For strain AG553, the SRA accession number is SRA188084; for strain AG601, the SRA accession number is SRA188071; for strain LL1210, the SRA accession number is SRR3503855.

### Proteomic analysis

Cell pellets for the four strains, wild-type *C. thermocellum*, AG553, AG601, and LL1210 were harvested at log phase and prepared for LC–MS/MS-based proteomic analysis. Briefly, proteins extracted via SDS, boiling, and sonic disruption were precipitated with trichloroacetic acid (TCA), cold acetone-washed, and pelleted as previously described [35]. The acetone-washed protein pellet was resolubilized in urea and treated with dithiothreitol and iodoacetamide to reduce and block disulfide bonds prior to digestion with sequencing-grade trypsin (Sigma-Aldrich). Following proteolysis, tryptic peptides were salted, acidified, and filtered through a 10 kDa MWCO spin column (Vivaspin 2; GE Healthcare) and quantified by BCA assay (Pierce).

For each LC–MS/MS run, 25 µg of peptides were loaded via pressure cell onto a biphasic MudPIT column [36] for online 2D HPLC separation (strong-cation exchange and reversed-phase) and concurrent analysis via nanospray MS/MS using a hybrid LTQ-Orbitrap XL mass spectrometer (Thermo Scientific) operating in data-dependent acquisition (one full scan at 15 k resolution followed by 10 MS/MS scans in the LTQ, all one µscan; monoisotopic precursor selection; rejection of analytes with an undecipherable charge; dynamic exclusion = 30 s). Eleven salt cuts (25, 30, 35, 40, 45, 50, 65, 80, 100, 175, and 500 mM ammonium acetate) were performed per sample run with each followed by 120 min organic gradient to separate peptides [37].

Resultant peptide fragmentation spectra (MS/MS) were searched against the *C. thermocellum* 1313 proteome database concatenated with common contaminants and reversed sequences to control false-discovery rates using MyriMatch v.2.1 [38]. Peptide spectrum matches (PSM) were filtered by IDPicker v.3 [39] using a peptide-level FDR of <1 % per sample run and assigned matched-ion intensities (MIT) based on observed peptide fragment peaks. PSM MITs were summed on a per-peptide basis, and only those uniquely and specifically matching a particular protein were moved onto subsequent analysis with InfernoRDN [40]. Peptide intensity distributions were log2-transformed, normalized across biological replicates by LOESS, and standardized by median absolute deviation and median centering across samples as suggested. Peptide abundance data were then assembled to proteins, scaled appropriately, and outliers removed (RRollup). Protein abundances were then filtered to maintain at least two values in at least one replicate set and missing values imputed using a random distribution of low-level values using Perseus (http://www.perseus-framework.org). Protein abundances were then compared across strains to identify proteins with differential abundance patterns.

## Additional files

10.1186/s13068-016-0528-8 Residual and products concentration from 50 g/L cellobiose during the fermentation. The strains LL1149 grown on minimal medium in bioreactor with pH regulation. The final ethanol titer was 14.38 g/L and the yield was 0.33 g_EtOH_/g_glu eq_.
10.1186/s13068-016-0528-8 End residual and products concentration from 95 g/L cellulose during the bioreactor fermentation. The strains grown on minimal medium in bioreactor with pH regulation. Error bars represent one standard deviation, n = 3. This is data for the end point after 303 h of incubation.
10.1186/s13068-016-0528-8 List of mutations among the strains AG553, AG601 and LL1210 of *Clostridium thermocellum.* *SNV* single nucleotide variation; *CDS* coding region.
10.1186/s13068-016-0528-8 Proteomic data of strains wild-type, AG553, AG553, AG601 and LL1210 of *Clostridium* *thermocellum*.

## Abbreviations

ADH: alcohol dehydrogenase
ALDH: aldehyde dehydrogenase
BSA: bovine serum albumin
CBP: consolidated bioprocessing
GAPDH: glyceraldehyde 3-phosphate dehydrogenase
MOPS: 3-morpholino-propane-1-sulfonic acid
PFK: phosphofructokinase
SNV: single nucleotide variation
